# Seroprevalence and Distribution of Contagious Bovine Pleuropneumonia in Ethiopia: Update and Critical Analysis of 20 Years (1996–2016) Reports

**DOI:** 10.3389/fvets.2017.00100

**Published:** 2017-06-28

**Authors:** Nejash Abdela, Nesradin Yune

**Affiliations:** ^1^School of Veterinary Medicine, College of Agriculture and Veterinary Medicine, Jimma University, Jimma, Ethiopia

**Keywords:** contagious bovine pleuropneumonia, distribution, Ethiopia, seroprevalence, trans-boundary animal disease

## Abstract

Contagious bovine pleuropneumonia (CBPP), infectious and highly contagious diseases of cattle in Africa, is the only bacterial disease in the OIE list A diseases. This severe respiratory disease of cattle is the second most important transboundary animal disease in Africa after rinderpest. CBPP is caused by *Mycoplasma mycoides* subsp. *mycoides* SC (small colony bovine biotype) and it is endemic disease in many African countries including Ethiopia. This paper systematically reviews prevalence report at herd and individual animal level for the last 20 years (1996–2016) with main aim of making comprehensive document regarding the seroprevalence and distribution of CBPP in Ethiopia. The paper is therefore helpful in knowing the past and current disease status and also to forecast the future and possible prevention option in the country. So far, the seroprevalence that ranges from 0.4 to 96% was reported from different export quarantine centers and production areas in Ethiopia. The reported seroprevalence is significantly associated with different agro-ecology of the country and the highest was reported from lowland in which 40% of livestock population was kept. The recent seroprevalence studies report from different areas of the country also indicated as CBPP is posing a major threat to cattle production in many parts of the country, thereby causing considerable economic losses through morbidity and mortality. This disease also causes restriction on the trade of animals and animal products internationally and accounts for a loss of over 8.96 million US dollars per year in Ethiopian situation. Thus, a great attention should be given both at production areas and the quarantine stations as its occurrence may affect the export earnings of the country, thereby threatening the livelihood of pastoralists and national economy of the country.

## Introduction

Ethiopia is a resourceful country bestowed with estimated largest livestock population in Africa. The livestock sector has a significant role in socioeconomic activities of the country and contributes much to the national economy (1). The contribution of livestock to the national economy particularly with regard to foreign currency earnings is through exportation of live animals, meat, skin, and hides (2, 3). However, development of this sector is hampered by different constraints. The most important constraints are widespread endemic diseases including viral, bacterial, and parasitic infestation (4–10). The other important bottleneck for the development of this sector include lack of appropriate disease control policy, lack of appropriate veterinary services, and lack of attention from government (6–8, 10–13). Among the health constraints, infectious disease like contagious bovine pleuropneumonia (CBPP) is considered to be one of the most economically important and major problem for Ethiopian livestock development (14–16).

Contagious bovine pleuropneumonia, also known as cattle lung disease, is one of the most important infectious and highly contagious diseases of cattle in Africa (17–19). The causative agent of CBPP is *Mycoplasma* mycoides subsp. *mycoides*, SC (small colony, bovine biotype) (20). The Pan African programme for the Control of Epizootics (PACE) identified CBPP as the second most important transboundary disease in Africa, after Rinderpest (21). Following the eradication of rinderpest from Africa, CBPP has become the disease of prime concern in terms of epizootics that affect cattle in the continent (20). It is estimated that annual losses due to CBPP amount to 38.81 million US dollars in 12 endemically infected sub-Saharan African countries (21). On account of its transmissibility and economic impacts, CBPP is now recognized as a priority transboundary disease and has thus been categorized as the only bacterial disease in the OIE list A diseases (22). CBPP is transmitted by direct contact between infectious and susceptible cattle and is characterized by its variable course and insidious nature (23).

The disease affects the respiratory tract of cattle and characterized by fever, anorexia, dyspnea, polypnea, cough, and nasal discharge (17, 24). All ages of cattle are susceptible, but young cattle develop joint swelling rather than lung infections. Many cattle show no disease signs despite being infected (25) and chronically infected animals might act as carriers and sources of infections (17).

This respiratory disease is considered as great economic importance to cattle keepers because of its high mortality rate, production loss, increased production cost due to cost of disease control, loss of weight and working ability, delayed marketing, reduced fertility, loss due to quarantine, loss of cattle trade, and reduced investment in livestock production (21, 26). Regarding the Ethiopian situation, CBPP has been causing significant economic losses on the livestock sector and the national economy. It accounts for a loss of over 8.96 million US dollars per year (27). Thus, over the last decades, the country has lost a substantial market share and foreign exchange earnings due to frequent bans by the Middle East countries (28).

Recent studies conducted in Western part of Ethiopia (16), Northern Ethiopia (29), Southern Ethiopia (30), Southwest Ethiopia (31), and different regions of the country (32) showed that CBPP is posing a major threat to cattle production in many parts of the country, thereby causing considerable economic losses through morbidity and mortality and demanding for serious attention from the concerned body. Furthermore, CBPP has been reported from different export quarantine centers in the country (33–35) signifying that CBPP remain a threat to livestock export market and may reduce the investment in livestock production. Studies undertaken so far in Ethiopia reported seroprevalence that range from 0.4% from export from export quarantine centers (15, 34) to 96% from Western Gojjam (36).

Despite aforementioned situation of CBPP in the country, there is scarcity of well-documented information. Therefore, the main objective of this paper was to make comprehensive document on seroprevalence and distribution of CBPP in the country. The paper systematically reviews prevalence report at herd and individual animal level for the last 20 years (1996–2016) and highlighted prevention options and future prospects.

## Historical Perspective and General Aspects of CBPP in Ethiopia

Epizootic diseases have threatened cattle since their domestication. Two of them played a prominent role in the last three centuries, namely rinderpest and CBPP threatening the livelihood of whole populations and hindering international cattle trade. However, while rinderpest has been successfully eradicated worldwide (37), CBPP is still present in many African countries including Ethiopia.

Although its origin is not documented, from a historical perspective CBPP was a disease of Europe and Asia (38). It was known in Europe in 1773 and it reached nearly worldwide distribution in the nineteenth century through the cattle trade (39). Through the application of restrictions to the movement of cattle, as well as test and slaughter policies combined with compensation for livestock keepers, CBPP has been eradicated from Australia, Europe, Asia, and America. However, such policies are difficult to apply in most African countries because of pastoralism, lack of economic resources, and fragmented veterinary services (40, 41). As a result, the disease remains endemic in Africa particularly in tropical and subtropical regions (West, Central, East, and parts of Southern Africa) of the continent (20, 40). Its incidence began to decline in Africa by the 1970s during the late 1980s and 1990s; however, this disease increased in prevalence in endemic areas and re-emerged in some African and European countries that had been CBPP free (19).

It is a prominent cattle disease in Africa, where outbreaks of the disease reported from 20 countries in 2006, with the highest number of cases in Ethiopia, Angola, and Cameroon (42). Over the last few years, major CBPP epidemics have been experienced in Eastern, Southern, and West Africa. Currently, it affects 27 countries in Africa with an estimated annual cost of US$2 billion (43). A total of 2,719 outbreaks were reported in Africa between 1995 and 2002 (21).

Regarding the situation in east Africa in general and Ethiopia in particular, there is a suggestion that CBPP was introduced into East Africa from India by the army of field Marshal Napier when he invaded Ethiopia in 1867–1868 (14). Countries in East Africa reported 66% of the total outbreaks (58% in Ethiopia and Tanzania and 8% in other countries in the region) (21). Ethiopia is one of east African countries in which CBPP is endemically maintained all over the country with 25% morbidity and more than 10% mortality (14, 21). A total of 583 outbreaks of CBPP were reported between 1995 and 2002 in Ethiopia in which highest outbreaks (187) were reported in 1998 (21). In general, CBPP has been causing significant economic losses on the agricultural sector and the national economy of Ethiopia. Over the last decades, the country has lost a substantial market share and foreign exchange earnings due to frequent bans by the Middle East countries (28). It accounts for a loss of over 8.96 million US dollars per year (27).

## Seroprevalence of CBPP and its Distribution in Ethiopia

According to Teklue et al. (29) and Mamo (31), the seroprevalence of CBPP is associated with the agro-ecology, and there is significant difference in different agro-ecology. It was found to occur at high rate, almost two times higher, in wet season of the year as compared with dry season (44). In addition, difference between month of the year has been indicated with highest seropositivity on July (0.7%) and lowest on June (0.1%) (15). Furthermore, a significantly higher seroprevalence was found in animals in the lowland than those in the highland and mid highland agro-ecology (31). This is major threat to the country as 40% of livestock population was kept under the pastoral lowland (45). Ethiopia has diverse agro-climatic conditions that can be broadly divided into highlands (1,500 m above sea level) 39% and lowlands (1,500 m below sea level) 61%. The lowlands, which are commonly referred to as “pastoral areas,” are found in the Eastern, South-Eastern, and Southern parts of the country. Moreover, Ethiopia is a tropical African country in which mobile pastoralism is dominant in the arid and semi-arid areas in the eastern, northeastern, and southeastern parts of the country (46). This practice facilitates the transmission of the disease from one herd or area to another and the establishment of the disease in the country (47).

**Figure 1:**
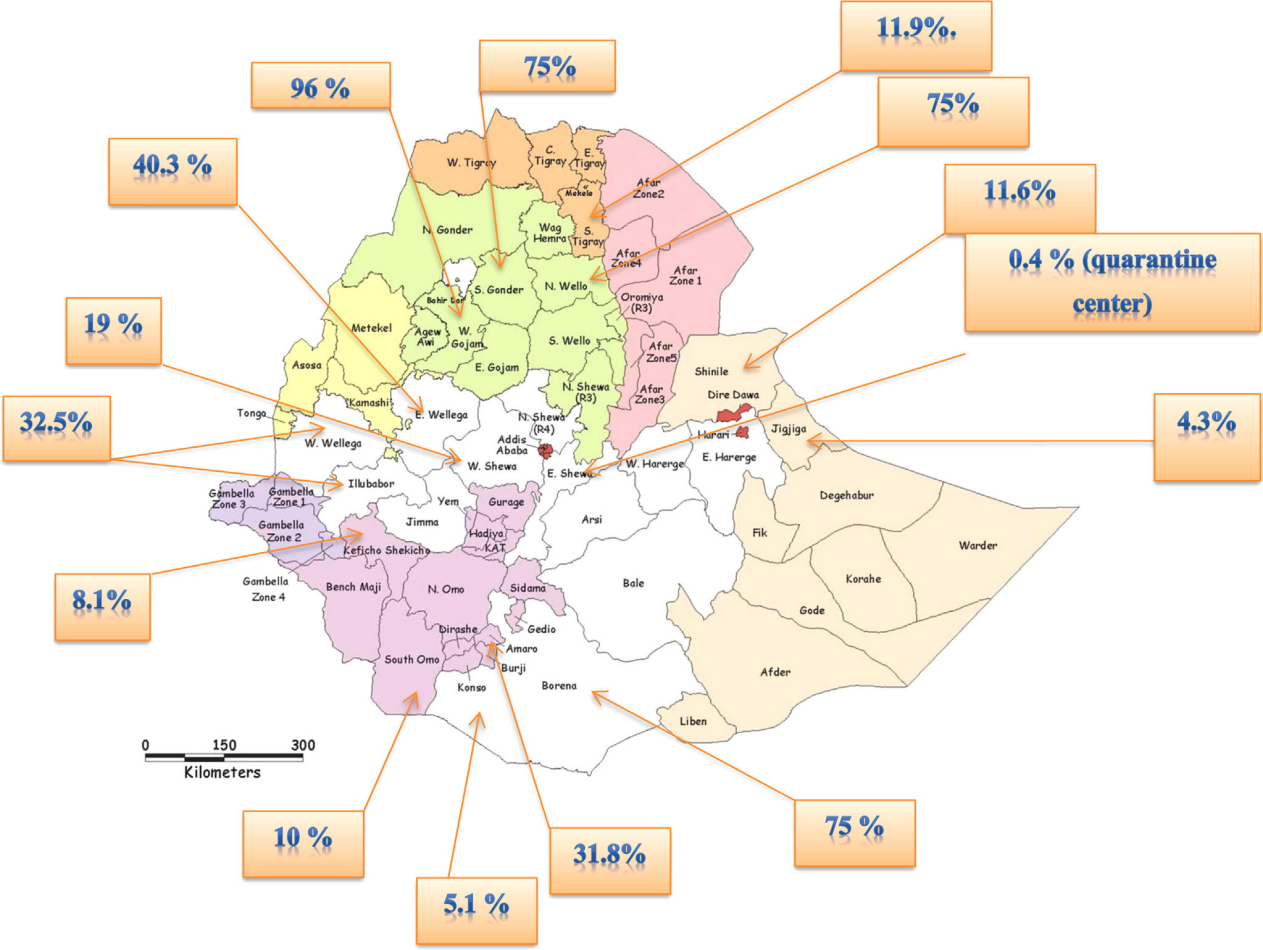
The current distribution of contagious bovine pleuropneumonia in different areas of Ethiopia.

Even though there is a paucity of research conducted on CBPP in the country, the disease is widespread and considered as one of the most important cattle diseases and impediments to livestock development in Ethiopia (20, 35, 48). Studies undertaken on CBPP so far revealed the existence of the disease in different parts of the country with prevalence that range from 0.4% (from bull at finishing phase for export in East Shewa zone that brought from Borena pastoral area) (15) to 96% in Western Gojjam (36). The current distribution of CBPP in different areas of Ethiopia is shown in Figure 1.

Gizaw (49) conducted serological study in two zones of the Somali Regional State (Jijiga and Shinille) in six districts. A total of 793 serum samples were tested from 56 herds and reported average seroprevalence of 30.4% in 17 herds with at least 1 infected animal per herd. The herd level prevalence reported was higher in Shinille zone (35.1%) than in Jijiga zone (21.1%). On the other hand, the individual level seroprevalence of the respective zones was 11.6 and 4.3%, which were much lower than herd prevalence. The highest herd seroprevalence was observed in Mieso (100%) followed by Qabribeyah (75%) and Afdem (71.4%) districts.

The study conducted by Kassaye and Molla (33) examined 3,111 cattle sera by using competitive enzyme-linked immunosorbent assay (c-ELISA) during a period of 2010–2011 and reported a 4% seroprevalence of CBPP at different export quarantine centers in and around Adama namely, Bekero, Dera, Jogo, and Kedir. The highest seroprevalence was reported from Bekero export quarantine farm (4.7%) and the lowest from Kedir (2.5%). Furthermore, they indicated as CBPP threat for Ethiopian livestock export market and a well-established disease in Borana and Bale areas, where the animals originated.

Another seroprevalence study from export quarantine centers by Birhanu (11) investigated CBPP at Adama-Modjo Livestock Export Industry in five Private beef animals’ Exporter Enterprises located in and around Adama. This study examined a total of 4,321 apparently healthy bulls for the prevalence of Anti body against CBPP by using 3ABC ELISA from November 2013 to May 2014 and reported the overall seroprevalence of 8.00%. Those bulls included in their seroprevalence investigation were come from Borena, Arsi, and Bale areas. From Borana out of 857 bulls examined, 61 are found to be positive with seroprevalence of 10.4%. On the other hand, among 1,432 bulls originated from Bale, 128 are found to be positive with seroprevalence of 8.90%. Furthermore, the lowest prevalence they report was from those bulls that were originated from Arsi with prevalence of 7.70% among 2019 bull investigated. Moreover, the highest was reported from Jordan Feedlot (22.7%) whereas the lowest seroprevalence was reported from Seyoum Feedlot (1.40%).

Ebisa et al. (30) also conducted seroprevalence study from November 2014 to April 2015 in Amaro special district of SNNP region to determine the seroprevalence of CBPP and to assess the potential risk factors for the occurrence of the disease. In their study, total of 400 sera were examined from 4 peasant associations (Jello, Kelle, Golbe, and Gamule) for the presence of specific antibodies against *M. mycoides* subspecies mycoides small colony type by using c-ELISA. Out of 400 sera examined, 127 animals were appeared to be positive and the overall seroprevalence of CBPP was reported to be 31.8%. Moreover, they have reported the highest CBPP seroprevalence (58%) from Gamule peasant association and the lowest seroprevalence (7%) from Jello peasant association of Amaro special district of SNNPR.

Teklue et al. (29) investigated CBPP from December 2012 to May 2013 in the four districts (Alamata, Raya Azebo, Ofla, and Endamehoni) of southern zone of Tigray region, Northern Ethiopia. A total of 384 sera samples examined, 36 sera were test positive to CFT giving an overall prevalence of 11.9%. Moreover, they have reported highest seroprevalence from Alamata district (28%) and lower 0 (0%) from Ofla and Endamehoni Districts.

Alemayehu et al. (15) conducted seroprevalence of CBPP for 1 year period from January 2011 to December 2011 by using c-ELISA on 40 batches of bulls admitted in 20 different feedlots facilities. A total of 38,187 apparently health bulls that were originated from Borena pastoral area and are on finishing stage for export in East Shewa zone are included in this study. Out of the total 40 batches tested for the presence of antibodies, 25 (62.5%) of them contained at least 1 seropositive bull and the overall seroprevalence reported was 0.4% which is relatively lower than the report of other researchers. However, the bulls used for the study was vaccinated against CBPP. At batches level, highest prevalence reported was from feedlots found in Dera, Mekie, Awash 7 killo, Nahmaled, and Adami Tulu sites (100%) whereas the lowest was recorded in feedlots found in Modjo and Awash Melkasa sites. Furthermore, at individual animal level, the highest seropositivity was indicated in Mekie (1.1%), and the lowest was indicated in Moddjo site (0.0%). Moreover, this study reported the highest CBPP prevalence in herd size >1,000 which is 93.8% and concluded that number of seropositive animals increases as the herd size increases.

Another seroprevalences study conducted by Dele et al. (34) in cattle originated from Borana at export quarantine centers in Adama revealed 0.4% prevalence out of 3,777 cattle investigated. The two studies (15, 34) on the animal from the same origin revealed the same result. However, the later one is conducted on apparently healthy animals intended for export to Egypt market that are not vaccinated for the CBPP. Moreover, another serological study in Borena zone revealed 5.1% prevalence by Issa (50) and 74% prevalence by Roger and Yigezu (51).

Atnafie et al. (35) conducted seroprevalence study from October 2014 to April 2015 to determine seroprevalence of CBPP among apparently healthy bulls to be slaughtered at selected abattoirs in Bishoftu and from export oriented feedlots around Adama. Total of 384 animals from abattoirs and 1,086 animals from feedlots were examined for the presence of antibody against CBPP using c-ELISA and about 30 (7.8%) animals from abattoirs and 65 animals (5.9%) from feedlots were found to be positive.

Furthermore, recently Daniel et al. (16) conducted seroprevalence study on CBPP from November 2013 to March 2014 in selected districts of three Western Oromia Zones (Western Shoa, Horro Guduru Wollegga, and Eastern Wollegga). In their study, total of 386 sera were examined for the presence of specific antibodies against *Mycoplasma mycoidesmycoides* small colony (MmmSC) by using a c-ELISA and reported an overall seroprevalence of 28.5%. Moreover, they indicated highest seroprevalence (40.3%) in Gobbu Sayyo district of Eastern Wollegga Zone, while the lowest seroprevalence (5.7%) in Horro district of Horro Guduru Wollegga Zone. The seroprevalence in Bako Tibbo of Western Shoa zone was 19.0%.

Another more recent seroprevalence study conducted in southwest Ethiopia by Mamo (31) investigated seroprevalence and associated risk factor from October 2015 to August 2016 by using c-ELISA. The total of 384 cattle in Gimbo district of Keffa zone Southern nation’s nationalities and people’s regional sate of Ethiopia was examined. The overall seroprevalence reported was 8.1% with the highest seroprevalence from lowland and followed by mid highland.

In general at the country level, CBPP seroprevalence studies have been conducted in different localities of the country. However, there is a great variation of reports from different areas this variation in the prevalence reported could be due to difference in agro-ecological system, animal management, production system, population density, and the types of tests used to evaluate the seroprevalence (16, 30). Bellini et al. (52) showed the specificity of CFT (threshold +1:10) to be 98% and sensitivity to be 63.79%. A c-ELISA test has undergone evaluation and is possible to apply at animal level (for interpretation) and compared with CF test, the c-ELISA has equal sensitivity and greater specificity. The c-ELISA is an individual test but you can aggregate the results and therefore interpret it at herd, and it is easier to perform than the CF test but its performance characteristics have not yet been fully assessed (22). The validation c-ELISA tests that have been carried out in several African and European countries have indicated true specificity of the c-ELISA to be at least 99.9% and the sensitivity of the c-ELISA and the CF test are similar (22). The antigen in CFT is a suspension of MmmSC, previously checkerboard titrated and used at a dose of two complement fixing units. A c-ELISA is based on a monoclonal anti-MmmSC antibody, named Mab 117/5 (22).

The seroprevalence report of CBPP from 1996 to 2016 from different areas of Ethiopia along with its distribution is summarized in Table 1.

**Table 1:** Seroprevalence reports of contagious bovine pleuropneumonia from different locations in Ethiopia from 1996 to 2016.

Reference	Study duration	Diagnostic test used	Location (study area)	Sample size	Prevalence (%)
(51)	November 1995 to May 1996	CFT	North Omo	374	29
			Konso S.D.	59	46
			Dirashe S.D.	70	32

(36)	1996	CFT	Western Wellega	360	75
			Western Gojam	80	96
			Southern Gondar	60	75
			Northern Wello	60	75
			Northern Shewa	100	100
			Southern Tigray	20	54
			Borena	370	75

(53)	November 1996 to May 1997	CFT	Western Ethiopia (Ilu Ababor and Wellega)		32.5

(54)	1996–1997	CFT and competitive enzyme-linked immunosorbent assay (c-ELISA)	Western Gojjam	2,140	9.1

(55)	November 2000 to April 2001	CFT	West Wellega	50 herds	4 (herd Prevalence)

(49)	2003–2004	c-ELISA	Jijiga zone	140	4.3
			Shinille zone	653	11.6

(50)	2003–2004	ELISA and CFT	Oromia (Borena zone)		5.1

(33)	October 2010 to March 2011	c-ELISA	Quarantine centers in and around Adama	3,111	4

(34)		CFT	Quarantine centers in and around Adama	3,777	0.4

(29)	December 2012 to May 2013	CFT	Tigray (southern zone)	384	11.9

(11)	November 2013 to May 2014	3ABC ELISA	Adama-Modjo Livestock Export Industry	4,321	8.00

(15)	January 2011 to December 2011	c-ELISA	Feed lot at East Shewa zone (animal originated from Borena zone)	38,187	0.4

(44)	–	c-ELISA	Dassenech district of South Omo Zone	68	10

(30)	November 2014 to April 2015	c-ELISA	SNNP (Amaro district)	400	31.8

(35)	October 2014 to April 2015	c-ELISA	Bishoftu	384	7.8
			Adama	1,086	5.9

(16)	November 2013 to March 2014	c-ELISA	Western Shoa	100	19
			Horro Guduru Wollegga	70	5.7
			Eastern Wollegga	216	40.3

(31)	October 2015 to August 2016	c-ELISA	SNNP	384	8.1
			Kefa Zone		

## Prevention and Future Prospects of CBPP

Transmission of CBPP requires direct contact between infectious and susceptible cattle (23). Therefore, strategies for CBPP control and eradication can rely on four broad approaches that aim to reduce effective animal contact: stamping out, cattle movement control, vaccination, and antimicrobial treatment (21, 56–58).

Stamping out involves slaughter of all CBPP-infected and in-contact cattle. This approach was used in combination with movement restriction to eradicate CBPP from the UK, North America, and Western Europe (59). Stamping out has been termed as the simplest and surest way to control and eradicate CBPP. However, stamping out has far reaching socioeconomic effects (60). Consequently, it is recommended that stamping out should be a strategy of last resort to be used in critical epidemiological situations such as in the case of outbreaks in a free area or the surveillance zone (of a sanitary cordon) or on major trade routes. It can also be introduced at a later stage of the campaign after substantial reduction of CBPP incidence such that the incidence is approaching 0 (61).

The application of movement restrictions and stamping out do not appear practically feasible to be applied in sub-Saharan Africa due to pastoral nature of cattle husbandry and resource limitations. Therefore, the levels of movement control consistent with sustainable pastoral livelihoods are unlikely to have a major impact on CBPP prevalence in the future (23). Thus, the control of CBPP has relied on preventive immunoprophylaxis using live-attenuated cultures of the causative agent.

The OIE recommends T1 vaccine strain for vaccination against CBPP. It is generally accepted that the protection offered by the vaccine wanes after 12 months (62) but may last for more than 1 year (63). The vaccine is sufficiently avirulent but can cause severe post-vaccinal adverse reactions in some breeds (64). In general, the successful eradication of CBPP can eliminate any future control cost of vaccination, treatment, quarantine, and movement control, which benefit producers and the nation. Control of CBPP is, therefore, important as a way to salvage the losses and increase the incomes of cattle owners (21).

All effective CBPP vaccines have been based upon live versions of the disease causing MmmSC, either attenuated or not. Currently, the only vaccines in use are live vaccine derived from the T1 strain (T1/44 and T1SR) of live-attenuated *M. mycoides* subsp. mycoides SC and attenuated through repeated passage in embryonated eggs before production in artificial growth media. Unfortunately, these vaccines are characterized by poor and variable efficacy, with only between 30 and 60% of vaccinated animals being protected. In some situations, the T1/44 vaccine induces a good immunity, especially when herds are revaccinated annually, in which case the level of protection exceeds 85%. Induced immunity is short lived, particularly for the T1SR strain, requiring revaccination at least on a yearly basis. Either systemic or local adverse reactions are common, particularly for the T1/44 strain. Within 2–4 weeks following injection, an invading edema develops known as the “Willems” reaction. The incidence of these reactions varies from one area to another. The reversion to virulence of the T1/44 vaccine has also been observed when it was serially passaged by endobronchial intubation resulting in the development of lesions of CBPP in animals that were infectious to in-contact animals. This suggests that animals given the currently used vaccines (T1/44 and T1SR) subcutaneously could be reservoirs for MmmSC and infect other animals in areas previously free of CBPP (65).

## Conclusion

Contagious bovine pleuropneumonia is economically important infectious and highly contagious disease of cattle characterized by variable course and insidious nature. The recent seroprevalence studies from different areas of Ethiopia witnessed as this severe respiratory disease is posing a major threat to livestock industry of the country. At the country level, seroprevalence studies have been conducted in different localities of the country both at production area and the quarantine stations. However, there is a great variation of reports from different areas that range from 0.4 to 96%. Furthermore, the reported seroprevalence is significantly associated with agro-ecology of the country and the highest was reported from lowland in which 40% of livestock population of the country was kept. Thus, it requires great attention both at production area and the quarantine stations as its occurrence may cause restriction on the trade of animals and animal products internationally, affecting the export earnings of the country, thereby threatening the livelihood of pastoralists and national economy. Moreover, effective vaccination policy needs to be considered as it is the only realistic method of choice for control of CBPP in the country with movement control if possible.

## Author Contributions

All authors listed have made substantial, direct, and intellectual contribution to the work and approved it for publication.

## Conflict of Interest Statement

The authors declare that the research was conducted in the absence of any commercial or financial relationships that could be construed as a potential conflict of interest.
